# Evaluation of the NIH Toolbox Cognition Battery across Healthy Agers and Mild Cognitive Impairment among Spanish‐Speaking Adults Aged 65 and Older: Results from the ARMADA Study

**DOI:** 10.1002/alz70857_107793

**Published:** 2025-12-26

**Authors:** Emily H Ho, Maya Ventura, Berivan Ece, Tatiana Karpouzian‐Rogers, Sandra Weintraub, Jennifer J. Manly, Richard C. Gershon

**Affiliations:** ^1^ Northwestern University Feinberg School of Medicine, Chicago, IL, USA; ^2^ Northwestern University, Evanston, IL, USA; ^3^ Mesulam Center for Cognitive Neurology & Alzheimer's Disease, Chicago, IL, USA; ^4^ Department of Neurology, Gertrude H. Sergievsky Center, Taub Institute for Research on Alzheimer's Disease and The Aging Brain, Columbia University Medical Center, New York, NY, USA

## Abstract

**Background:**

Older Hispanic/LatinX populations are at increased risk compared with older White adults to develop dementia, yet few studies have characterized their performance on standardized neuropsychological batteries such as the NIH Toolbox Cognition Battery (NIHTB‐CB). We investigated the differences of NIHTB‐CB tests and composites in a 100% Spanish‐speaking and Hispanic sample of healthy agers, compared to those diagnosed with amnestic Mild Cognitive Impairment (aMCI) and mild dementia of the Alzheimer's Type (mDAT).

**Method:**

As part of the larger Advancing Reliable Measurement in Alzheimer's Disease and Cognitive Aging (ARMADA) Study, we assessed 125 healthy agers (87.2% female; *M_age_* = 73.2, *SD_age_* = 4.99), 123 with an aMCI diagnosis (63.4% female; *M_age_* = 76.50, *SD_age_* =  6.30), and 28 with mild DAT (64.3% female; *M_age_* = 75.60, *SD_age_* =  7.62). The NIHTB‐CB was administered by trained examiners included Oral Reading Recognition (ORR), Picture Vocabulary (PV), List Sort Working Memory (LSWM) Picture Sequence Memory (PSM), Pattern Comparison Processing Speed (PC), Dimensional Change Card Sort (DCCS), Flanker Inhibitory Control and Attention (Flanker) tests. In a series of ANOVAs, we investigated the differences in unadjusted scale scores, adjusting for age, education, sex as a biological variable, and study site.

**Result:**

Compared to healthy agers, those with aMCI scored significantly lower on all NIHTB‐CB measures and composites, with high standardized mean differences for LSWM (Diff = ‐11.73, 95% CI=[‐14.88, ‐8.57]), PCPS (Diff = ‐8.62, 95% CI=[‐12.44, ‐4.8]), DCCS (Diff = ‐8.19, 95% CI=[‐10.95, ‐5.43]), and PV (Diff = ‐8.02, 95% CI=[‐10.64, ‐5.39]). Observed differences between the healthy ager and mild DAT group were even higher, as expected: LSWM (Diff = ‐14.29, 95% CI=[‐20.55, ‐8.02]), PCPS (Diff = ‐17.71, 95% CI=[‐24.25, ‐11.17]), DCCS (Diff = ‐11.34, 95% CI=[‐16.27, ‐6.41]), and PV (Diff = ‐12.57, 95% CI=[‐16.93, ‐8.22]). The NIHTB‐CB differentiated between aMCI and mild DAT only on PCPS and PV. These differences were independent of sex or age effects, except for PCPS, where an increase in age predicted lower performance.

**Conclusion:**

Nearly all measures within the NIHTB‐CB significantly differentiate between healthy controls and individuals with aMCI, and healthy agers and individuals with mDAT among older Spanish‐speaking adults.

